# Impact of Telehealth on the Delivery of Prenatal Care During the COVID-19 Pandemic: Mixed Methods Study of the Barriers and Opportunities to Improve Health Care Communication in Discussions About Pregnancy and Prenatal Genetic Testing

**DOI:** 10.2196/38821

**Published:** 2022-12-05

**Authors:** Caitlin G Craighead, Christina Collart, Richard Frankel, Susannah Rose, Anita D Misra-Hebert, Brownsyne Tucker Edmonds, Marsha Michie, Edward Chien, Marissa Coleridge, Oluwatosin Goje, Angela C Ranzini, Ruth M Farrell

**Affiliations:** 1 Obstetrics and Gynecology and Women's Health Institute Cleveland Clinic Cleveland, OH United States; 2 Research Innovation and Education Cleveland Clinic Lerner College of Medicine Cleveland Clinic Cleveland, OH United States; 3 Center for Patience Experience Cleveland Clinic Cleveland, OH United States; 4 Department of Internal Medicine Cleveland Clinic Community Care Cleveland Clinic Cleveland, OH United States; 5 Department of Obstetrics and Gynecology Indiana University Indianapolis, IN United States; 6 Department of Bioethics Case Western Reserve University Cleveland, OH United States; 7 Genomic Medicine Institute Cleveland Clinic Cleveland, OH United States; 8 Department of Obstetrics and Gynecology MetroHealth Medical Center Cleveland, OH United States; 9 Center for Bioethics Cleveland Clinic Cleveland, OH United States

**Keywords:** prenatal health care delivery, health care communication, telehealth, access to health care, COVID-19, pregnancy

## Abstract

**Background:**

The COVID-19 pandemic brought significant changes in health care, specifically the accelerated use of telehealth. Given the unique aspects of prenatal care, it is important to understand the impact of telehealth on health care communication and quality, and patient satisfaction. This mixed methods study examined the challenges associated with the rapid and broad implementation of telehealth for prenatal care delivery during the pandemic.

**Objective:**

In this study, we examined patients’ perspectives, preferences, and experiences during the COVID-19 pandemic, with the aim of supporting the development of successful models to serve the needs of pregnant patients, obstetric providers, and health care systems during this time.

**Methods:**

Pregnant patients who received outpatient prenatal care in Cleveland, Ohio participated in in-depth interviews and completed the Coronavirus Perinatal Experiences-Impact Survey (COPE-IS) between January and December 2021. Transcripts were coded using NVivo 12, and qualitative analysis was used, an approach consistent with the grounded theory. Quantitative data were summarized and integrated during analysis.

**Results:**

Thematic saturation was achieved with 60 interviews. We learned that 58% (35/60) of women had telehealth experience prior to their current pregnancy. However, only 8% (5/60) of women had used both in-person and virtual visits during this pregnancy, while the majority (54/60, 90%) of women participated in only in-person visits. Among 59 women who responded to the COPE-IS, 59 (100%) felt very well supported by their provider, 31 (53%) were moderately to highly concerned about their child’s health, and 17 (29%) reported that the single greatest stress of COVID-19 was its impact on their child. Lead themes focused on establishing patient-provider relationships that supported shared decision-making, accessing the information needed for shared decision-making, and using technology effectively to foster discussions during the COVID-19 pandemic. Key findings indicated that participants felt in-person visits were more personal, established greater rapport, and built better trust in the patient-provider relationship as compared to telehealth visits. Further, participants felt they could achieve a greater dialogue and ask more questions regarding time-sensitive information, including prenatal genetic testing information, through an in-person visit. Finally, privacy concerns arose if prenatal genetic testing or general pregnancy conversations were to take place outside of the health care facility.

**Conclusions:**

While telehealth was recognized as an option to ensure timely access to prenatal care during the COVID-19 pandemic, it also came with multiple challenges for the patient-provider relationship. These findings highlighted the barriers and opportunities to achieve effective and patient-centered communication with the continued integration of telehealth in prenatal care delivery. It is important to address the unique needs of this population during the pandemic and as health care increasingly adopts a telehealth model.

## Introduction

Health care delivery has changed dramatically as a result of the COVID-19 pandemic, with telehealth as a major resource to maintain health care access during this time [1,2]. Telehealth had been developing into an accepted modality to deliver health care prior to the COVID-19 pandemic, with data emerging about health care quality, patient-provider communication, and patient satisfaction with this alternative approach to in-person care [3-5]. Although the application of telehealth in obstetric care is not new, the integration of telehealth within prenatal care had not been broadly implemented in many practices across the United States prior to the pandemic [6]. The pandemic accelerated its implementation and, in doing so, shed light on some of the most significance benefits, drawbacks, and challenges of its rapid implementation across diverse patient populations, in addition to the need to study outcomes using this modality [6-9].

Telehealth was a particularly important prenatal care strategy to maintain health care access while helping to prevent viral exposure to pregnant patients and health care providers, as well as communities [6-8]. Early on in the pandemic, it was established that pregnant patients infected with SARS-CoV-2 had increased risks of intensive care unit admission and death [10]. Thus, it was critical to use telehealth to help avoid exposure, since there was limited data about the best approaches for infection prevention and management among the pregnant population. The need for regular and timely access to care was additionally significant; prenatal care delivery involved multiple time-sensitive and potentially complex health care decisions. Prenatal genetic screens and diagnostic tests are examples, which are time-sensitive with respect to the gestational age at which the testing windows open and close [11]. A delay in access to information about these tests, or use of these tests, can have major implications for the outcome of the pregnancy [12]. Because of factors such as these, telehealth was rapidly implemented across average-risk and high-risk patients, and at different gestational ages [13-15]. Yet, there was limited opportunity to understand the impact of uptake on patient experience and key markers of health care quality during the pandemic, a time in which patients had a new and additional set of informational priorities about the prevention and management of SARS-CoV-2 infection in the health care discussion.

Prenatal care is complex, even at the best of times, and clear communication from clinicians and comprehension by patients can be challenging. The increased use of non–face-to-face communication modalities (eg, telephone and virtual video visits) during the pandemic has introduced greater complexity as well as opportunities and risks to interpersonal communication. Little is currently known about the impact of virtual visits in novel contexts such as a pandemic. To address this gap, we conducted a mixed methods study to better understand patients’ lived experiences of virtual prenatal care.

## Methods

### Study Design

This study was developed as a mixed methods study to explore emerging concepts and themes as they relate to obstetric health care delivery and patient experience during the pandemic.

### Recruitment

Participants were 18 years of age or older, were English speaking, had a viable intrauterine pregnancy, and received outpatient obstetric care. We recruited pregnant women at outpatient centers within the Cleveland Clinic and MetroHealth health care systems between January and December 2021.

Participants were contacted by means of a recruitment letter. The letter invited patients, who met the inclusion criteria and were interested in sharing their knowledge and opinions of decision‐making surrounding prenatal testing in light of the COVID‐19 pandemic, to contact the research team. Recruitment was structured to seek input from 2 groups of patients who represented patients at different significant time points in pregnancy. One group included patients in the first trimester of pregnancy to capture prenatal care needs, preferences, and experiences at the onset of pregnancy and prenatal care delivery (Group 1). A second group included patients in the second trimester, who had already considered or undergone prenatal genetic screening or diagnostic testing at the time of the interview (Group 2). Recruitment continued until thematic saturation in interviews was reached.

### Data Collection

After an informed consent process, each participant participated in a telephone interview to maintain consistency with the health care systems’ recommendations for social distancing and patient contact for research purposes at the onset of the pandemic. Interviews were conducted by a member of the research team using a semistructured interview guide, which contained questions about knowledge and opinions on COVID-19, prenatal care delivery during the pandemic, accessing information about prenatal genetic testing during the pandemic, accessing prenatal genetic testing during the pandemic, health care system resources to support patients, and demographic and reproductive history. This guide was developed in conjunction with content experts in obstetrics, clinical genetics, medical decision‐making, patient experience, and maternal‐fetal medicine. With the participants’ permission, the interviews were audio recorded and then transcribed verbatim for analysis.

Data were also collected using the self-administered Coronavirus Perinatal Experiences-Impact Survey (COPE-IS) [16]. The COPE-IS is a newly developed survey to understand the experiences of pregnant women during the COVID-19 pandemic. It has not been psychometrically tested at this time [17-19]. The survey was administered to participants after the telephone interview was completed to assess both the events and circumstances of women’s lives as new or expectant mothers during the time of the global pandemic. The survey was administered via REDCap Survey accessed on a computer or mobile device, or if the patient preferred, a hard copy was mailed with a stamped envelope to send back to the study team once completed.

### Statistical Analysis

Qualitative analysis was approached as an iterative and progressive process of data immersion, coding, memoing, and theme identification, which is an inductive process consistent with the grounded theory [20,21]. We identified content domains and categories in transcripts to create a coding tree used to organize the data. A companion codebook was created to serve as a reference for the analysis. The coding and analysis processes were led by 2 members of the study team (RMF and CGC) using NVivo (version 12; QSR International). The research team held weekly meetings to review data coding and memoing, and identify themes. Themes identified were contextualized with information about the trimester of pregnancy, gravity/parity, and previous pregnancies. Data from the COPE-IS and demographic information were summarized as frequency and mean. Quantitative data were summarized and integrated during analysis.

### Ethical Considerations

This study was reviewed in advance, approved, and monitored by the Cleveland Clinic Institutional Review Board (IRB number 20-1333). The Institutional Review Board approved a waiver of remote written consent from participants via DocuSign, a 21 Code of Federal Regulations Part 11–compliant electronic signature platform. All identifying information inadvertently disclosed by the study participants during the interviews were deleted from the original data file, and all study participant data were deidentified. Additionally, all participants received a US $50 gift card after the completion of the interview and COPE-IS.

## Results

### Participant Demographics

Thematic saturation was achieved with 60 interviews. Of the 60 patients, 30 were in their first trimester (Group 1) and 30 were in their second trimester (Group 2). The average age of the participants was 31 (SD 4.28) years. Moreover, this was the first pregnancy for 22 (37%) women, and 17 (28%) were considered to have an advanced maternal age (Table 1). Of the 60 women, 35 (58%) had telehealth experience prior to their pregnancy; however, 54 (90%) participated in only in-person visits during this pregnancy.

**Table 1:** Participant demographics.

Demographic		Value (N=60)
Age (years), mean (SD)		31.1 (4.28)
**AMA^a^ status, n (%)**		
	Non-AMA (<35 years)	43 (72)
	AMA (≥35 years)	17 (28)
**Race, n (%)**		
	White	49 (82)
	Black	3 (5)
	Asian	3 (5)
	Multiracial	2 (3)
	Declined to answer	3 (5)
**Reproductive history, n (%)**		
	Primigravida	22 (37)
	Multigravida	38 (63)
**Trimester of pregnancy**		
	1st trimester	30 (50)
	2nd trimester	30 (50)
**Prior telehealth experience**		
	Yes	35 (58)
	No	20 (33)
	Unsure	5 (8)
**Visit type during this pregnancy**		
	In-person visit only	54 (90)
	Virtual visit only	1 (2)
	Hybrid, used both in-person and virtual visits	5 (8)

^a^AMA: advanced maternal age.

### COPE-IS

Almost all of the participants completed the COPE-IS (59/60, 98%), and among these, 59 (100%) indicated feeling very well supported by their primary care provider, 31 (53%) reported feeling moderately to highly concerned about the impact of COVID-19 on their child’s health, and 17 (29%) reported the single greatest stress due to COVID-19 was its impact on their child (Table 2). All data from the COPE-IS are presented in Multimedia Appendix 1.

**Table 2:** Coronavirus Perinatal Experiences-Impact Survey results.

Question and response				Value (N=59), n (%)
**How well are you currently being supported by your primary prenatal care provider(s)?**				
	Very well supported			59 (100)
	Somewhat well supported			0 (0)
	Not very well supported			0 (0)
**Has the support you receive from your prenatal care changed due to the COVID-19 outbreak?**				
	Significantly worsened			0 (0)
	Somewhat worsened			1 (2)
	No change			49 (83)
	Somewhat improved			6 (10)
	Significantly improved			3 (5)
**Do you have any concerns about your child’s health as a result of the COVID-19 outbreak?**				
	No			24 (41)
	**Yes (score)**			35 (59)
		1, no concern	0 (0)	
		2	0 (0)	
		3	4 (11)	
		4	6 (17)	
		5	10 (29)	
		6	7 (20)	
		7, highly concerned	8 (23)	
**In general, how distressed are you about your own COVID-19–related symptoms or potential illness? (score)**				
	1, no distress			18 (31)
	2			6 (10)
	3			10 (17)
	4			6 (10)
	5			13 (22)
	6			5 (9)
	7, highly distressed			1 (2)
**How has the COVID-19 outbreak changed your stress levels or mental health?**				
	Worsened them significantly			6 (10)
	Worsened them moderately			30 (51)
	No change			20 (34)
	Improved them moderately			3 (5)
	Improved them significantly			0 (0)
**Overall level of stress related to the COVID-19 outbreak (score)**				
	1, nothing			6 (10)
	2			9 (15)
	3			15 (25)
	4			13 (22)
	5			11 (19)
	6			2 (3)
	7, extreme			3 (5)
**What is the single greatest source of stress due to the COVID-19 outbreak right now? (check only one)**				
	Impact on your child			17 (29)
	Health concerns			13 (22)
	Impact on family members (eg, elderly parents)			9 (15)
	Financial concerns			5 (9)
	General well-being due to social distancing and/or quarantine			5 (9)
	Impact on society			4 (7)
	I am not stressed			3 (5)
	Impact on your partner			2 (3)
	Access to baby supplies (eg, formula, diapers, and wipes)			1 (2)
	Impact on your community			0 (0)
	Impact on close friends			0 (0)
	Access to food			0 (0)
	Access to mental health care			0 (0)
	Stress about other aspects (open field)			0 (0)

### Themes

Qualitative analysis identified the following primary themes: (1) establishing patient-provider relationships that supported shared decision-making, (2) accessing the information needed for a shared decision-making process, and (3) using technology effectively to foster discussions during the COVID-19 pandemic. These themes and example quotes are presented in Table 3.

**Table 3:** Themes and example quotes.

Theme	Quotes
Establishing patient-provider relationships that supported shared decision-making	“Although like if she’s giving…if you’re giving somebody really terrible news, I think you would want them…I mean, it’s probably [easier] to be in-person just to make sure the person understands everything you know?” [Group 1, Participant #5] “I would always put the face-to-face above because of the fluidity and the conversation that happens when you're there in-person.” [Group 1, Participant #21] “For me, it’s not like she spelled everything out for me and went over every little thing. She just said, ‘This was available to you. Do you know what you want to do?’ and, ‘Do you have any questions about it?’ And, I really didn’t at the time. I know they talked about the book they provided, and I looked up some of my own stuff online and heard stuff from other people. So, I just came to my own conclusions. But, for some people who might be really worried about prenatal testing or are on the fence about it, an in-person might be better to talk about it. They do feel a little different [in-person vs VV conversations]. It almost feels awkward in a way. That’s just what I got. It feels wrong. It felt a little more rushed, and I don’t know if that was because she was running a few minutes late so then she was running late to another one afterwards, or, she really didn’t have a lot of questions for me and I didn’t have a lot of questions for her. But yeah, it did have a little of a rushed feeling.” [Group 2, Participant #2] “I was grateful to be able to have a pretty long in-person conversation with my OB/GYN about genetic testing on several occasions prior to the actual conversation with the genetic counselor. That made me feel at ease that I had the space and the time to have a pretty long conversation and ask a lot of questions on a couple of different occasions. So I’m grateful that have that the first time, be in-person and have the space to ask a lot of questions.” [Group 2, Participant #26] “I think the benefits… I just… like that one-on-one interaction. Especially now being a stay at home mom with very little outings … if I had a virtual appointment he’s [her son] all over me. So, it’s a little distracting, where I feel like I get…I have more of a clear head for a one-on-one interaction without him around.” [Group 2, Participant #14] “For me personally like someone who's gonna be best involved in intimate care, I just feel more comfortable meeting with the person.” [Group 1, Participant #14]
Accessing the information needed for a shared decision-making process	“More of a personal touch…I think…that’s one of the hardest things right now is if you have to separate from people…so that’s kinda nice to have actual interaction face-to face….and then to me it just feels like...it is more secure, you are able to ask questions….I don’t have to worry about a screaming child in the back so I don’t have any distractions…I just like in-person visits better.” [Group 1, Participant #2] “I think there could be benefits for the right people who are comfortable enough and confident enough and asking the right questions over, over video and things like that.” [Group 1, Participant #11] “The downside, I keep saying, I think like it’s those questions that are asked I feel like are different then when I’ve done a virtual visit. I went to one virtual visit and it was very quick and then I ended up seeing my doctor in-person a few months later and the way questions were asked and things were discussed were completely different feeling when I did a virtual visit.” [Group 2, Participant #18] “I forget to ask questions cause I find the virtual visits a little bit awkward and instead of being an in-person thing…” [Group 2, Participant #22]
Using technology effectively to foster discussions during the COVID-19 pandemic	“For some reason, it didn’t connect for a while. So, I didn’t think that … I thought I lost her. Like, I thought I almost missed the appointment because it wouldn’t connect or it was being goofy.” [Group 1, Participant #8] “I like to talk to people in-person. I don’t like to be over the phone or people in my house. So, going inside the hospital is much better for me.” [Group 2, Participant #25] “I do as long as I can find a space to have that conversation. Usually those conversations happen while I am at work. So I’ve had to remove myself and find a private…go sit in my car. But I am not concerned about my information being, you know the security of it.” [Group 2, Participant #26] “Hardest thing for me was finding a quite space while the kids were home to have them [the appointments].” [Group 2, Participant #18]

#### Establishing Patient-Provider Relationships as the Basis for Shared Decision-Making During the COVID-19 Pandemic

The strength of the patient-provider relationship and trust were important themes. Participants expressed different opinions about the quality of the patient-provider relationship when engaging in in-person or telehealth visits. Some participants characterized telehealth visits as a more convenient and safer way to receive prenatal care during the pandemic. One participant who experienced telehealth during pregnancy stated:

It’s a minimizing exposure thing. I think it's very empowering to the client to have to, or the patient to have to take their own blood pressure, it helps with their accountability and their engagement in their care to have to be able to, Doppler, their fetal heart rate and all that stuff. So, I think that would be a benefit. Also, being in the comfort of your own home is kind of nice.

Group 1, Participant #19

For some, the level of preference regarding telehealth was a function of prior experience with pregnancy.

I guess because of all of that, because of the convenience, because of COVID, I now feel comfortable with my second one [pregnancy] to do some visits virtually and don't really have concerns that I wouldn’t see her as often.

Group 2, Participant #13

In addition, some did not see a significant difference in how health care communication may unfold in this setting.

I’m sure they would give you the same information either way, I mean the information is the same, how it is being delivered is different.

Group 2, Participant #27

However, most of the participants indicated that in-person visits were “more personal” and had a greater potential for “establishing a rapport” with the provider.

The drawback [to virtual visits] is definitely the personal touch, just talking to somebody through a screen. So you’re not actually there and maybe feeling like, if you were worried about something you wouldn’t feel the same emotions I guess coming from your doctor.

Group 2, Participant #2

For some, this modality hindered the development of trust with their provider.

I don’t feel the same trust that you can build with a doctor if you are face to face with them versus on a tele-call.

Group 2, Participant #27

This trust was particularly important during pregnancy and with respect to the nature of the issues and decisions that are often made in this setting. Compared to other clinical settings, prenatal care was described as a “personal” visit with a unique “level of intimacy” needed when discussing issues of reproduction and pregnancy.

I do think it's easier to build a relationship with your provider in-person, which I think for this kind of thing, for pregnancy, is important, at least for me.

Group 1, Participant #11

Trust and relationship building were important in themselves, but also contributed to the quality of patient-provider communication. Participants reflected that they were accustomed to in-person visits and the kinds of patient-provider interactions that take place in the consultation or exam room. The transition to a virtual visit modality was unfamiliar to many, and for this reason, they reported that this was a very different health care delivery experience for them. This difference was due, in part, to their prior experience of in-person visits and having a level of comfort with discussions in this format. One participant who reflected on her preference for in-person visits stated:

I feel like in-person I’d definitely feel more at ease talking about it then over the phone or wherever virtually...

Group 1, Participant #16

This was also due to an overall lower familiarity with using a video conferencing platform for prenatal care prior to the pandemic, particularly at the beginning of a new patient-provider relationship. One participant stated:

During a virtual visit I think it will be harder for me I guess … just like you breaking out of the shell like, you know, on a Zoom call to bring something up.

Group 2, Participant #5

In-person discussions were described as interactions in which “conversation is more natural than like through Zoom or whatever…FaceTime” [Group 2, Participant #18]. This was the case even if there was baseline familiarity with using these platforms for nonmedical reasons.

I think it’s just easier to communicate. You know we’ve all had one million Zoom meetings at this point. So, you know, I think we’ve all gotten better at communicating through the virtual methods. But I also think that there’s just something that can’t be replaced about, you know, questions that come up in the moment. And it’s sort of easier to talk in-person…

Group 1, Participant #4

Overall, participants reflected on the need to learn about how best to use telehealth to obtain the same level of experience they expected and were accustomed to with in-person visits.

The need for in-person interaction was even more important in situations that called for an accurate, time-sensitive, and patient-centered discussion for care planning. One participant who reflected on a specific connection with her health care provider now that her pregnancy was at increased risk because of maternal age stated:

I like to look my doctor in the eye and I feel like I’m older in pregnancy now. So, I feel like I just…I need that one-on-one.

Group 1, Participant #6

This type of interaction was even more important when there was a chance of a difficult conversation, including in cases when the patient received information about a potential problem with the pregnancy.

It’s probably easier to be in-person just to make sure the person understands everything you know?

Group 1, Participant #5

An in-person dynamic also played a role in the setting of conversations regarding care decisions that may be complex (eg, multiple different options or steps in a testing algorithm), entrenched in patient values, or potentially sensitive. One participant described the nature of prenatal genetic testing decisions and the need to have accurate information from their health care provider about the choices as follows:

I think it’s [prenatal genetic testing] a touchy subject and there's a lot of different, you know, advice out there about having that knowledge and the benefits and also cons of it. So, I think having that conversation in-person it made me feel more comfortable.

Group 1, Participant #10

This level of communication was also seen as a benefit to help mitigate the uncertainty that can be associated with these decisions. One participant stated:

For me personally, I knew I wanted to do the genetic testing. So, I don’t think it would have made a huge difference but I can see, for some people again, who might be unsure of what the right choice is for them, I think again in-person, it just makes that communication easier to talk about different options of why one may or may not choose genetic testing.

Group 1, Participant #18

Some participants noted that nonverbal communication was a key component of successful conversations and that in-person interactions were an important opportunity for the provider to assess how to approach a conversation or the level of the patient’s understanding and for the patient to know how to interpret the provider’s information.

I think it’s important for them [the healthcare provider] to just see what you look like and, you know, how you're doing, how you're reacting to things, like I think there's a lot that goes into nonverbal communication and that's harder to do when you’re not in person.

Group 1, Participant #18

Participants discussed potential opportunities for issues to be minimized. One such opportunity was for health care provider education in order to be certain that a telehealth visit would provide an equivalent level of patient experience and health care communication that fostered a shared decision-making process. One participant stated:

I also had conversations with the genetic counselors and she was very good at the skill of virtual visits so she allowed that kind of space. But I don’t think that is characteristic of every healthcare professional. She did settle in and have a long conversation and a long phone conversation as well and sat through my repetitive questions. Because she was good at the skill, a virtual appointment was okay. But I don’t think that style of conversation is indicative of every clinical provider.

Group 2, Participant #26

Another suggested opportunity was a hybrid model that alternated telehealth visits with in-person visits. This approach would allow for the patient to establish a relationship and obtain additional information in a conventional health care format as needed for prenatal care decision-making.

So, maybe at this point in the game, I would be okay with doing a virtual visit as long as I knew maybe next month we’d meet in person. I wouldn’t want to do virtual visits my entire pregnancy but I would be okay with doing it every now and then.

Group 2, Participant #4

The success of such a hybrid model would be related to an individualized approach that would be dependent upon the estimated gestational age of the pregnancy with associated prenatal care milestones and the needs of the patient.

#### Accessing the Information Needed for a Shared Decision-Making Process During the COVID-19 Pandemic

Underlying these comments was a concern that key prenatal care discussions may be limited and that telehealth could impede access to information about care choices. Many participants suggested that they might obtain more information about their obstetric care during an in-person visit. The preference for in-person discussion primarily pertained to the ability to have a “more natural” and dynamic discussion in which there was a shared decision-making conversation with the provider.

When you’re face to face there’s a little more openness and ability to think on your feet as to what other questions or areas of concern that you can bring up with the doctor.

Group 1, Participant #21

Participants noted that in-person visits allowed them to ask questions to obtain the information they needed, while attending to their reproductive and medical history needs as well.

One participant for whom this was her first pregnancy stated:

This is my first pregnancy. There is a lot that I don’t know. So, I find the flow of the conversation moves a million times better in an in-person conversation and there is more space to stop and think when someone asks, ‘Do you have any questions?’ I find it that the full conversation becomes truncated over the phone and there is less time for thinking and working through questions or letting information arrive in a conversation … I find that more information gets volunteered, details get talked through in an in-person conversation versus a phone conversation … The drawbacks [of a virtual visit], I see, are a less comprehensive visit, less comprehensive care, less opportunity for more information to come to light in the conversation.

Group 2, Participant #26

For this participant, her absence of prior experience with pregnancy made her more uncertain about what questions she should ask, with concerns that if she did not initiate the question, she may not receive information that was important to her. In addition, some participants expressed preferences for modalities in which they learned best, including how they received the information and were able to retain and integrate it into health care decisions. One participant stated:

I feel like it helps to be in-person because of the fact that it’s easier to retain the information than over a video call.

Group 1, Participant #30

Thus, there were questions about whether and how the telehealth visit may affect their ability to obtain the information they desired and process that information in a way that would support informed decision-making.

For several participants, in-person visits were preferred due to the chance to mentally prepare for the visit and think through questions and goals for the discussion.

If I am running around the house and then I log on I’m probably not thinking about the visit as much or like the questions that I want to ask her… And sometimes I think- maybe I think of more questions cause I’m sitting in the waiting room and I’m actually think about it, rather than like running around the house and I’m logging on real quick for an appointment.

Group 2, Participant #8

Participants also spoke of concerns about “distractions” in the home environment that may negatively impact the ability to obtain information about their health care and potentially decrease the level of communication that allows for a shared decision-making process.

There is so much going on with being at home during the pandemic, that it is difficult to find the focus to concentrate on the discussion at hand.

Group 1, Participant #25

#### Using Technology Effectively to Foster Discussions During the COVID-19 Pandemic

Participants also discussed concerns about technology-related factors associated with virtual visits, something they did not have to worry about during an in-person visit. One participant discussed her difficulties with obtaining a clear connection with her health care provider as follows:

There’s just technical difficulties and usually it’s on like an internet connection type level, sometimes the calls a little choppy or sometimes patients have a difficult time getting on, you know, just some basic, technical issues.

Group 2, Participant #17

Participants also expressed concerns about how technological or internet issues could distract them from focusing on what was important to them during the conversation with their provider. One participant stated:

Maybe someone who doesn’t know how to use the platform to do the appointment might get a little confused and frustrated. I will say, when I did my first appointment virtually, it wasn’t a problem, but my doctor was late. And, I was like, ‘Oh shoot. Wait…do I pick up the phone and call the front desk and ask if we need to reschedule or is she just running behind?’ Usually, when you’re in the waiting room or in the room waiting for the doctor to come in, which happens all the time, you know they are eventually going to come in. So, I feel like when you’re waiting on a call like that and it’s just a blank screen you’re like, ‘Shoot. Is something wrong with my computer or is it like she’s running late?’ I feel like there is just more question with that.

Group 2, Participant #2

For this participant, the uncertainty about missing the appointment or having a failed connection caused her to be more distracted during the visit, with less time to prepare questions that she aimed to ask during the visit.

Participants identified concerns about privacy and the level of privacy that could be acquired during the visit. One level of concern pertained to safety and security associated with using a mobile or other personal device for health care, particularly applicable in the context of discussing topics relevant to reproductive history. One participant spoke of her reluctance to use internet-enabled devices for private discussions as follows:

I feel like personally we all, everybody, knows that the government kind of watches us and tracks us through our phones and everything.

Group 1, Participant #30

Issues of privacy also pertained to a participant’s ability to access a location for the telehealth visit when it took place outside of a consultation or exam room in the clinic, especially for those participants who worked outside of the home during the pandemic.

I don’t have a private office. So, sometimes even calling to make a doctor’s appointment is like a little… you know… I go out to my car or I try to get into a conference room to make that call just because that’s a personal thing. You don’t want your coworkers hearing, especially if you’re calling to make your eight week appointment saying, ‘Hey, I just took a pregnancy test. When can I come in?’ So yeah, if I wasn’t working from home, I would definitely want to go into the doctor’s office in-person.

Group 2, Participant #3

While some participants could find a private space at work or in their car, others struggled with whether and where they would have the resources for the kinds of discussions they needed with their health care provider. Privacy was also an issue for those participants who remained at home during the pandemic, several of whom raised concerns about access to a private space in the home for conversations about potentially highly sensitive and personal topics related to the pregnancy and their reproductive health. Some participants commented as follows:

I can see where someone would feel uncomfortable depending on where their setting is.

Group 2, Participant #27

I don’t have other kids in the house. I don’t have other family. It’s just my husband and I in our home. So, I have privacy in our home to carry on those conversations. I don’t feel like I need to be in a doctor’s office to have a safe conversation with somebody or private conversation with someone. So, I can see how for other people that that might not be their situation. They may not have as much privacy at home.

Group 2, Participant #4

## Discussion

### Principal Findings

Prenatal care delivery is uniquely complex given the complexity and time-sensitive nature of the decisions that need to be made regarding maternal and fetal health. The rapid and robust introduction of telehealth has added an additional layer of complexity, with numerous variables that could interfere with effective patient-provider discussions. Our study demonstrates that there is a spectrum of opinions regarding pregnant patients’ perceptions of the effects of telehealth on health care quality and satisfaction during the COVID-19 pandemic. Although telehealth has been available for many years, increase in its use during the pandemic had a wide-scale impact on obstetric health care providers and patients. Studies conducted both prior to and during the pandemic demonstrated that telehealth visits may improve access to health care, decrease childcare needs, eliminate transportation and parking costs, reduce office wait times, and, most importantly, minimize exposure to COVID-19 [22-25]. In response, a series of authors have established protocols for integrating telehealth and hybrid models into prenatal care episodes [26-28]. Yet, a parallel discussion has suggested that increased satisfaction and convenience observed with telehealth visits may not equate to the same levels of health care quality and patient-centered care as observed during in-person visits [29]. Data are emerging that some patients may prefer lower technology visits, such as via the telephone, over those that involve a video component, according to factors related to patient characteristics [30]. As health care increasingly adopts telehealth models, it is important to determine how systems will adapt these methods to diverse patient and provider populations with different knowledge, resources, and skills to utilize telehealth overall and in the unique setting of prenatal care, in which often complex and time-sensitive decisions with significant implications for obstetric outcomes must be made. In addition, it is important to understand how factors, such as demographics and the presence of stressors (eg, the impact of the COVID-19 pandemic on individuals and pregnant patients), may play a role in telehealth implementation and utilization.

The findings of this study are significant as we identified several additional issues, apart from those related to technology. One important theme we identified was a concern about the barriers to health care communication resulting from the conversion of in-person visits to telehealth encounters. These barriers have both clinical and ethical implications as access to accurate and patient-centered information is a component of health care quality. Technological issues, such as the ability to access and use mobile devices with the appropriate level of broadband internet, in addition to familiarity with telehealth platforms, are factors contributing to the digital divide [31]. Issues with technology may lead to a cascade of downstream implications for patient-provider communication. This may begin with how individuals in the health care discussion express informational priorities in addition to how they exchange and receive relevant information in health care discussions. Ultimately, the interaction by and among individuals in the clinical encounter can have ramifications for the medical options presented by providers and the health care decisions that patients make during pregnancy.

We identified several other issues in addition to those associated with using technology. These included the degree of effective communication, trust in the therapeutic relationship, and patient-centered care that they had expected for their prenatal care or had experienced in prior pregnancies. These factors all related to the ability to seek and acquire information in a way that supported an informed decision-making process about their prenatal care. These are factors that may also relate to the degree of impact experienced from the COVID-19 pandemic, as demonstrated by participant responses to the COPE-IS. In part, these barriers were attributed to patients’ unfamiliarity with differences in communication styles between telehealth and in-person visits, which patients were not aware of prior to the visits, and thus, they may not have had an opportunity to adapt their behaviors or actions accordingly. These findings echo observations of other researchers. For example, studies demonstrated that communication in telehealth visits is different from that in in-person visits in significant ways. Telehealth visits may be more physician-centered than patient-centered, characterized by a communication style driven by provider-centered behaviors that make assumptions about the patients’ interests and needs [29,32]. Telehealth visits have also been associated with less discussion about and orientation to agenda setting or additional patient concerns that come up during the visit, which represent aspects that are of key importance in the delivery of prenatal care [12]. In addition, there may be limited opportunities for patients to ask questions and relay their understanding of the key concepts of the conversation with the provider, which is a key aspect of patient-centered care [32]. These issues must be addressed for all patients, particularly those who face existing health care disparities and may face additional challenges that interfere with shared decision-making [31]. Notably, previous studies involved general internal medicine visits, raising the question of how the unique aspects of prenatal care delivery may be affected by variations in communication and patient-centeredness.

Our findings also bring to light novel issues of privacy with the integration of telehealth into reproductive health care. Prenatal care visits may address what patients may consider private, personal, and sensitive topics relating to parenthood and reproduction. Those discussions can be additionally salient when discussing prenatal genetic screening and diagnostic testing, in which issues related to heritable genetic risk factors, family history (eg, issues of paternity for the current pregnancy), disability, and pregnancy termination are discussed [12]. Participants raised concerns about prenatal telehealth relating to not only internet security, but also the ability to find a space in their home or workplace away from family, friends, or coworkers to have those conversations. This may be a particularly important factor among patient populations of lower socioeconomic status, where household crowding and housing instability are more common. In addition, home internet access may be unaffordable, requiring patients to access care in public libraries or other public venues. In turn, these concerns about privacy may have limited their ability to ask questions, provide responses, build trust, and engage in shared decision-making.

As telehealth continues to be integrated into prenatal care, it will be critical to discuss ways to prepare patients for some of the differences they may encounter between telehealth and in-person visits. These may include establishing resources, such as health care extenders, who can educate patients about the telehealth visit prior to their appointment, providing an orientation to the telehealth interface, and educating patients about differences they can expect from a virtual versus in-person visit. Though there was little opportunity to develop such strategies during the pandemic due to the urgent need to protect patients, especially pregnant patients, from exposure to SARS-CoV-2 infection, emerging data highlight the need for additional efforts to improve telehealth visits in future circumstances. Existing theoretical frameworks, such as interpersonal communication theory and symbol interaction theory, provided a basis for developing effective approaches to health care communication in telehealth applications [33-37]. It is important to contextualize these foundational theories with the perspectives of pregnant patients who can inform the best practices moving forward.

At the same time, it is also critical to educate obstetric providers about how to facilitate the visit in a way that is most supportive of patient-centered communication. It is important for health care providers to recognize that they may need to adjust how they conduct a telehealth visit compared to an in-person visit in response to patients’ familiarity, receptivity, and resources in order to optimize this format. This includes an awareness of how both verbal and nonverbal cues and information may differ in a telehealth visit compared to an in-person visit [33,37,38] and, in turn, the effect of those different modes on patients’ access to information and medical decision-making. In establishing these practices, it may be of benefit to utilize one or more of the developed approaches to improve communication in telehealth visits [38,39]. For example, the health literacy universal precautions approach “assumes that all patients are at risk for miscommunication and misunderstanding” [39-41]. Using this approach and reflecting, providers can prepare for telehealth visits with communication techniques that will support high-quality prenatal care and the skill set to rapidly transition between in-person and telehealth modalities quickly in a busy clinic. In developing the best practices moving forward, it is important to consider the diversity of pregnant patients’ needs and preferences. Existing protocols focus on prioritizing telehealth visits for encounters that do not involve a procedure that requires an in-person visit or for average-risk patients [28]. Yet, patients in this study suggested that visits in which important, complex, and time-sensitive discussions must be made (eg, discussions about prenatal genetic screening and diagnostic testing) are also significant events, for which some of the dynamics of an in-person visit would be of benefit. These findings call for additional research to understand how best to individualize a plan of in-person and telehealth visits for patients based on their resources, needs, and preferences, independent of reproductive history.

### Limitations

While our study provides insights into the clinical and ethical challenges with implementing telehealth, the findings should be contextualized with the limitations of this study. The study was based on patients from health care systems in Ohio that adopted telehealth protocols in similar ways during the pandemic. Nonetheless, it is possible that there were subtle differences in the ways in which the practices associated with them were implemented. Although we sought a broad demographic representation in our recruitment efforts, most participants were <35 years of age (72%), self-described White (82%), and from the same geographic area. As a result, our results, by design, are not meant to be generalizable. We acknowledge that other health care systems and geographic areas of the United States may have had other experiences or practices with respect to telehealth delivery. In our population, more than half of the participants had a telehealth experience prior to pregnancy in addition to having at least one telehealth visit during the current pregnancy. While our sample represented patients with different reproductive histories, our sample was limited in racial and ethnic representation. Despite these limitations, the study brings to light important findings for which further research is needed to elucidate about larger and more diverse patient populations.

### Conclusion

The variables that affect health care communication are complex factors that may differ based on in-person versus telehealth interactions. While telehealth was utilized as a mechanism to ensure timely access to prenatal care during the COVID-19 pandemic, it also comes with multiple challenges and opportunities to develop best practices around its continued integration into health care delivery. Our study speaks to the variability in patient perceptions of the utility and usability of telehealth for prenatal care delivery and the need to identify evidence-based approaches to individualize care. This includes education and strategies to support effective patient-centered communication so that patients can access the information and decision support needed to make the often complex, time-sensitive, and critical decisions that characterize prenatal health care. As health care communication is a key component of health care quality and patient safety, it is essential that we understand how to develop best practices around telehealth as its role in the delivery of prenatal care grows.

## Appendix

Multimedia Appendix 1 Overall and individual Coronavirus Perinatal Experience-Impact Survey data.

## Abbreviations

COPE-IS: Coronavirus Perinatal Experience-Impact Survey

## References

[1] Litchfield I, Shukla D, Greenfield S (2021). Impact of COVID-19 on the digital divide: a rapid review. BMJ Open.

[2] Eruchalu CN, Pichardo MS, Bharadwaj M, Rodriguez CB, Rodriguez JA, Bergmark RW, Bates DW, Ortega G (2021). The expanding digital divide: Digital health access inequities during the COVID-19 pandemic in New York City. J Urban Health.

[3] DeNicola N, Grossman D, Marko K, Sonalkar S, Butler Tobah YS, Ganju N, Witkop CT, Henderson JT, Butler JL, Lowery C (2020). Telehealth interventions to improve obstetric and gynecologic health outcomes: A systematic review. Obstet Gynecol.

[4] Lowery C (2018). Telehealth: A new frontier in ob/gyn. Contemporary OB/GYN.

[5] van den Heuvel JFM, Teunis CJ, Franx A, Crombag NMTH, Bekker MN (2020). Home-based telemonitoring versus hospital admission in high risk pregnancies: a qualitative study on women's experiences. BMC Pregnancy Childbirth.

[6] Spiegelman J, Krenitsky N, Syeda S, Sutton D, Moroz L (2020). Rapid development and implementation of a Covid-19 telehealth clinic for obstetric patients. NEJM Catalyst.

[7] No authors listed (2020). Implementing telehealth in practice. Obstet Gynecol.

[8] Zork NM, Aubey J, Yates H (2020). Conversion and optimization of telehealth in obstetric care during the COVID-19 pandemic. Semin Perinatol.

[9] Danylchuk N, Cook L, Shane-Carson K, Cacioppo C, Hardy M, Nusbaum R, Steelman SC, Malinowski J (2021). Telehealth for genetic counseling: A systematic evidence review. J Genet Couns.

[10] Pregnant and Recently Pregnant People: At Increased Risk for Severe Illness from COVID-19. Centers for Disease Control and Prevention.

[11] American College of Obstetricians and Gynecologists’ Committee on Practice Bulletins—Obstetrics, Committee on Genetics, Society for Maternal-Fetal Medicine (2020). Screening for fetal chromosomal abnormalities: ACOG Practice Bulletin, Number 226. Obstet Gynecol.

[12] Farrell RM, Pierce M, Collart C, Tucker Edmonds B, Chien E, Coleridge M, Rose SL, Perni U, Frankel R (2020). Making the most of the first prenatal visit: The challenge of expanding prenatal genetic testing options and limited clinical encounter time. Prenat Diagn.

[13] Aziz A, Zork N, Aubey JJ, Baptiste CD, D'Alton ME, Emeruwa UN, Fuchs KM, Goffman D, Gyamfi-Bannerman C, Haythe JH, LaSala AP, Madden N, Miller EC, Miller RS, Monk C, Moroz L, Ona S, Ring LE, Sheen J, Spiegel ES, Simpson LL, Yates HS, Friedman AM (2020). Telehealth for high-risk pregnancies in the setting of the COVID-19 pandemic. Am J Perinatol.

[14] Galle A, Semaan A, Huysmans E, Audet C, Asefa A, Delvaux T, Afolabi BB, El Ayadi AM, Benova L (2021). A double-edged sword-telemedicine for maternal care during COVID-19: findings from a global mixed-methods study of healthcare providers. BMJ Glob Health.

[15] Peahl AF, Powell A, Berlin H, Smith RD, Krans E, Waljee J, Dalton VK, Heisler M, Moniz MH (2021). Patient and provider perspectives of a new prenatal care model introduced in response to the coronavirus disease 2019 pandemic. Am J Obstet Gynecol.

[16] Thomason M, Graham A, VanTieghem M (2020). COPE: Coronavirus Perinatal Experiences - Impact Survey (COPE-IS). PhenX Toolkit.

[17] Kinser PA, Jallo N, Amstadter AB, Thacker LR, Jones E, Moyer S, Rider A, Karjane N, Salisbury AL (2021). Depression, anxiety, resilience, and coping: The experience of pregnant and new mothers during the first few months of the COVID-19 pandemic. J Womens Health (Larchmt).

[18] Werchan DM, Hendrix CL, Ablow JC, Amstadter AB, Austin AC, Babineau V, Anne Bogat G, Cioffredi L, Conradt E, Crowell SE, Dumitriu D, Fifer W, Firestein MR, Gao W, Gotlib IH, Graham AM, Gregory KD, Gustafsson HC, Havens KL, Howell BR, Humphreys KL, King LS, Kinser PA, Krans EE, Lenniger C, Levendosky AA, Lonstein JS, Marcus R, Monk C, Moyer S, Muzik M, Nuttall AK, Potter AS, Salisbury A, Shuffrey LC, Smith BA, Smith L, Sullivan EL, Zhou J, Thomason ME, Brito NH (2022). Behavioral coping phenotypes and associated psychosocial outcomes of pregnant and postpartum women during the COVID-19 pandemic. Sci Rep.

[19] Motrico E, Mateus V, Bina R, Felice E, Bramante A, Kalcev G, Mauri M, Martins S, Mesquita A (2020). Good practices in perinatal mental health during the COVID-19 pandemic: a report from task-force RISEUP-PPD COVID-19. Clínica y Salud.

[20] Strauss A, Corbin J, Denzin NK, Lincoln YS (1994). Grounded theory methodology: An overview. Handbook of Qualitative Research.

[21] Ryan GW, Bernard H (2003). Techniques to identify themes. Field Methods.

[22] Madden N, Emeruwa UN, Friedman AM, Aubey JJ, Aziz A, Baptiste CD, Coletta JM, D'Alton ME, Fuchs KM, Goffman D, Gyamfi-Bannerman C, Kondragunta S, Krenitsky N, Miller RS, Nhan-Chang C, Saint Jean AM, Shukla HP, Simpson LL, Spiegel ES, Yates HS, Zork N, Ona S (2020). Telehealth uptake into prenatal care and provider attitudes during the COVID-19 pandemic in New York City: A quantitative and qualitative analysis. Am J Perinatol.

[23] Holcomb D, Faucher MA, Bouzid J, Quint-Bouzid M, Nelson DB, Duryea E (2020). Patient perspectives on audio-only virtual prenatal visits amidst the severe acute respiratory syndrome coronavirus 2 (SARS-CoV-2) pandemic. Obstet Gynecol.

[24] Almuslim H, AlDossary S (2022). Models of incorporating telehealth into obstetric care during the COVID-19 pandemic, its benefits and barriers: A scoping review. Telemed J E Health.

[25] Wu H, Sun W, Huang X, Yu S, Wang H, Bi X, Sheng J, Chen S, Akinwunmi B, Zhang CJP, Ming W (2020). Online antenatal care during the COVID-19 pandemic: Opportunities and challenges. J Med Internet Res.

[26] Peahl AF, Smith RD, Moniz MH (2020). Prenatal care redesign: creating flexible maternity care models through virtual care. Am J Obstet Gynecol.

[27] Peahl AF, Zahn CM, Turrentine M, Barfield W, Blackwell SD, Roberts SJ, Powell AR, Chopra V, Bernstein SJ (2021). The Michigan plan for appropriate tailored healthcare in pregnancy prenatal care recommendations. Obstet Gynecol.

[28] Kern-Goldberger AR, Srinivas SK (2022). Obstetrical telehealth and virtual care practices during the COVID-19 pandemic. Clin Obstet Gynecol.

[29] Agha Z, Roter DL, Schapira RM (2009). An evaluation of patient-physician communication style during telemedicine consultations. J Med Internet Res.

[30] Alkureishi MA, Choo Z, Rahman A, Ho K, Benning-Shorb J, Lenti G, Velázquez Sánchez I, Zhu M, Shah SD, Lee WW (2021). Digitally disconnected: Qualitative study of patient perspectives on the digital divide and potential solutions. JMIR Hum Factors.

[31] Paige SR, Bunnell BE, Bylund CL (2022). Disparities in patient-centered communication via telemedicine. Telemed J E Health.

[32] Gordon HS, Solanki P, Bokhour BG, Gopal RK (2020). "I'm not feeling like I'm part of the conversation" patients' perspectives on communicating in clinical video telehealth visits. J Gen Intern Med.

[33] Bylund CL, Peterson EB, Cameron KA (2012). A practitioner's guide to interpersonal communication theory: an overview and exploration of selected theories. Patient Educ Couns.

[34] Elias N (1991). The Symbol Theory.

[35] Carter MJ, Fuller C (2016). Symbols, meaning, and action: The past, present, and future of symbolic interactionism. Current Sociology.

[36] Berger CR, Roloff ME, Stacks DW, Salwen MB, Eichhorn KC (2019). Interpersonal Communication. An Integrated Approach to Communication Theory and Research.

[37] Charmaz K, Belgrave LL, Cockerham W (2013). Modern Symbolic Interaction Theory and Health. Medical Sociology on the Move.

[38] Miller EA (2002). Telemedicine and doctor-patient communication: a theoretical framework for evaluation. J Telemed Telecare.

[39] Coleman C (2020). Health literacy and clear communication best practices for telemedicine. Health Lit Res Pract.

[40] Health Literacy Universal Precautions Toolkit. AHRQ.

[41] Coleman C, Hudson S, Pederson B (2017). Prioritized health literacy and clear communication practices for health care professionals. Health Lit Res Pract.

