# Supplementary material for: Impact of Telehealth on the Delivery of Prenatal Care During the COVID-19 Pandemic: Mixed Methods Study of the Barriers and Opportunities to Improve Health Care Communication in Discussions About Pregnancy and Prenatal Genetic Testing
Source: JMIR Form Res. 2022 Dec 5;6(12):e38821. doi: 10.2196/38821 (PMC9728023; doi:10.2196/38821)
Supplement: Multimedia Appendix 1 [file formative_v6i12e38821_app1.docx]

**Multimedia Appendix 1.** Overall and individual Coronavirus Perinatal Experience-Impact Survey data.

| Patient ID | How well are you currently being supported by your primary prenatal care provider(s)? | Has the support you receive from your prenatal care changed due to the COVID-19 outbreak? |
| --- | --- | --- |
|  | Very well supported (59, 100.0%)  Somewhat well supported (0, 0.0%)  Not very well supported (0, 0.0%) | Significantly worsened (0, 0.0%)  Somewhat worsened (1, 1.6%)  No change (49, 83.0%)  Somewhat improved (6, 10.2%)  Significantly improved (3, 5.0%) |
| G1-01 | Very well supported | No change |
| G1-02 | Very well supported | No change |
| G1-03 | Very well supported | No change |
| G1-04 | Very well supported | No change |
| G1-05 | Very well supported | No change |
| G1-06 | Very well supported | Somewhat improved |
| G1-07 | Very well supported | No change |
| G1-08 | Very well supported | No change |
| G1-09 | Very well supported | No change |
| G1-10 | Very well supported | Somewhat improved |
| G1-11 | Very well supported | No change |
| G1-12 | Very well supported | No change |
| G1-13 | Very well supported | No change |
| G1-14 | Very well supported | No change |
| G1-15 | Very well supported | No change |
| G1-16 | Very well supported | No change |
| G1-17 | Very well supported | No change |
| G1-18 | Very well supported | No change |
| G1-19 | Very well supported | No change |
| G1-20 | Very well supported | Somewhat worsened |
| G1-21 | Very well supported | No change |
| G1-22 | Very well supported | No change |
| G1-23 | Very well supported | No change |
| G1-24 | Very well supported | Significantly improved |
| G1-25 | Very well supported | No change |
| G1-26 | Very well supported | Somewhat improved |
| G1-27 | Very well supported | No change |
| G1-28 | Very well supported | No change |
| G1-29 | Very well supported | Significantly improved |
| G1-30 | Very well supported | No change |
| G2-01 | Very well supported | No change |
| G2-02 | Very well supported | No change |
| G2-03 | Very well supported | No change |
| G2-04 | Very well supported | No change |
| G2-05 | Very well supported | No change |
| G2-06 | Very well supported | No change |
| G2-07 | Very well supported | No change |
| G2-08 | Very well supported | No change |
| G2-09 | Very well supported | No change |
| G2-10 | Very well supported | No change |
| G2-11 | Very well supported | No change |
| G2-12 | Very well supported | No change |
| G2-13 | Very well supported | Somewhat improved |
| G2-14 | Very well supported | Somewhat improved |
| G2-15 | Very well supported | No change |
| G2-16 | Very well supported | No change |
| G2-17 | Very well supported | No change |
| G2-18 | Very well supported | Significantly improved |
| G2-19 | Very well supported | No change |
| G2-20 | Very well supported | No change |
| G2-21 | Very well supported | No change |
| G2-22 | Very well supported | No change |
| G2-23 | Very well supported | No change |
| G2-24 | Very well supported | No change |
| G2-25 | Very well supported | No change |
| G2-26 | Very well supported | No change |
| G2-28 | Very well supported | Somewhat improved |
| G2-29 | Very well supported | No change |
| G2-30 | Very well supported | No change |

| Patient ID | Do you have any concerns about your child's health as a result of the COVID-19 outbreak? | In general, how distressed are you about your own COVID-19 related symptoms or potential illness? |
| --- | --- | --- |
|  | No (24, 40.7%)  Yes (35, 59.3%)  1 No concern (0, 0.0%)  2 (0, 0.0%)  3 (4, 11.4%)  4 (6, 17.1%)  5 (10, 28.6%)  6 (7, 20.0%)  7 Highly concerned (8, 22.9%) | 1 No distress (18, 30.5%)  2 (6, 10.2%)  3 (10, 16.9%)  4 (6, 10.2%)  5 (13, 22.0%)  6 (5, 8.5%)  7 Highly distressed (1, 1.7%) |
| G1-01 | No | 1 No distress |
| G1-02 | Yes – 3 | 5 |
| G1-03 | Yes – 7 Highly concerned | 6 |
| G1-04 | Yes – 4 | 1 No distress |
| G1-05 | Yes – 5 | 3 |
| G1-06 | No | 1 No distress |
| G1-07 | No | 4 |
| G1-08 | Yes – 5 | 6 |
| G1-09 | Yes – 6 | 1 No distress |
| G1-10 | Yes – 7 Highly concerned | 5 |
| G1-11 | Yes – 6 | 5 |
| G1-12 | No | 1 No distress |
| G1-13 | No | 2 |
| G1-14 | Yes – 4 | 3 |
| G1-15 | Yes – 4 | 3 |
| G1-16 | Yes – 7 Highly concerned | 1 No distress |
| G1-17 | No | 1 No distress |
| G1-18 | Yes – 4 | 3 |
| G1-19 | Yes – 5 | 4 |
| G1-20 | No | 1 No distress |
| G1-21 | Yes – 5 | 5 |
| G1-22 | Yes – 7 Highly concerned | 1 No distress |
| G1-23 | Yes – 7 Highly concerned | 7 Highly distressed |
| G1-24 | Yes – 6 | 5 |
| G1-25 | No | 3 |
| G1-26 | No | 5 |
| G1-27 | No | 2 |
| G1-28 | No | 1 No distress |
| G1-29 | No | 5 |
| G1-30 | Yes – 7 Highly concerned | 5 |
| G2-01 | Yes – 6 | 5 |
| G2-02 | Yes – 5 | 4 |
| G2-03 | No | 1 No distress |
| G2-04 | Yes – 6 | 1 No distress |
| G2-05 | Yes – 5 | 2 |
| G2-06 | Yes – 3 | 4 |
| G2-07 | No | 6 |
| G2-08 | Yes – 3 | 1 No distress |
| G2-09 | Yes – 4 | 4 |
| G2-10 | No | 1 No distress |
| G2-11 | No | 2 |
| G2-12 | No | 5 |
| G2-13 | Yes – 5 | 4 |
| G2-14 | No | 1 No distress |
| G2-15 | Yes – 4 | 5 |
| G2-16 | No | 3 |
| G2-17 | Yes – 5 | 3 |
| G2-18 | Yes – 6 | 3 |
| G2-19 | Yes – 5 | 1 No distress |
| G2-20 | No | 2 |
| G2-21 | Yes – 7 Highly concerned | 6 |
| G2-22 | Yes – 6 | 5 |
| G2-23 | Yes – 5 | 1 No distress |
| G2-24 | No | 1 No distress |
| G2-25 | No | 2 |
| G2-26 | Yes – 3 | 3 |
| G2-28 | No | 3 |
| G2-29 | Yes – 7 Highly concerned | 6 |
| G2-30 | No | 5 |

| Patient ID | How has the COVID-19 outbreak changed your stress levels or mental health? | Overall level of stress related to the COVID-19 outbreak. |
| --- | --- | --- |
|  | Worsened them significantly (6, 10.2%)  Worsened them moderately (30, 50.8%)  No change (20, 33.9%)  Improved them moderately (3, 5.1%)  Improved them significantly (0, 0.0%) | 1 Nothing (6, 10.2%)  2 (9, 15.3%)  3 (15, 25.4%)  4 (13, 22.0%)  5 (11, 18.6%)  6 (2, 3.4%)  7 Extreme (3, 5.1%) |
| G1-01 | No change | 4 |
| G1-02 | Worsened them moderately | 4 |
| G1-03 | Worsened them moderately | 6 |
| G1-04 | Improved them moderately | 3 |
| G1-05 | Worsened them moderately | 4 |
| G1-06 | No change | 3 |
| G1-07 | No change | 4 |
| G1-08 | No change | 4 |
| G1-09 | Worsened them moderately | 3 |
| G1-10 | Worsened them moderately | 4 |
| G1-11 | No change | 4 |
| G1-12 | No change | 1 Nothing |
| G1-13 | No change | 1 Nothing |
| G1-14 | Worsened them moderately | 3 |
| G1-15 | Worsened them moderately | 5 |
| G1-16 | Worsened them moderately | 4 |
| G1-17 | Worsened them moderately | 5 |
| G1-18 | Worsened them moderately | 3 |
| G1-19 | Worsened them moderately | 3 |
| G1-20 | No change | 2 |
| G1-21 | Worsened them significantly | 5 |
| G1-22 | Worsened them moderately | 3 |
| G1-23 | Worsened them significantly | 7 Extreme |
| G1-24 | Worsened them significantly | 7 Extreme |
| G1-25 | No change | 2 |
| G1-26 | No change | 3 |
| G1-27 | Worsened them moderately | 5 |
| G1-28 | No change | 1 Nothing |
| G1-29 | Worsened them moderately | 2 |
| G1-30 | Worsened them moderately | 5 |
| G2-01 | Worsened them moderately | 5 |
| G2-02 | Worsened them significantly | 5 |
| G2-03 | No change | 2 |
| G2-04 | Worsened them moderately | 4 |
| G2-05 | Worsened them significantly | 7 Extreme |
| G2-06 | Worsened them moderately | 6 |
| G2-07 | Worsened them moderately | 5 |
| G2-08 | Worsened them moderately | 4 |
| G2-09 | Worsened them moderately | 3 |
| G2-10 | No change | 2 |
| G2-11 | No change | 1 Nothing |
| G2-12 | No change | 3 |
| G2-13 | Improved them moderately | 3 |
| G2-14 | Worsened them moderately | 2 |
| G2-15 | Worsened them moderately | 2 |
| G2-16 | No change | 2 |
| G2-17 | Worsened them moderately | 4 |
| G2-18 | Worsened them moderately | 3 |
| G2-19 | No change | 1 Nothing |
| G2-20 | Worsened them moderately | 4 |
| G2-21 | Worsened them moderately | 5 |
| G2-22 | No change | 5 |
| G2-23 | Worsened them moderately | 4 |
| G2-24 | No change | 3 |
| G2-25 | No change | 1 Nothing |
| G2-26 | Worsened them moderately | 3 |
| G2-28 | Improved them moderately | 2 |
| G2-29 | Worsened them significantly | 5 |
| G2-30 | Worsened them moderately | 3 |

| Patient ID | What is the single greatest source of stress due to the COVID-19 outbreak right now? (check only one) |
| --- | --- |
|  | Impact on your child (17, 28.8%)  Health concerns (13, 22.0%)  Impact on family members (e.g. elderly parents) (9, 15.3%)  Financial concerns (5, 8.5%)  General well-being due to social distancing and/or quarantine (5, 8.5%)  Impact on society (4, 6.8%)  I am not stressed (3, 5.1%)  Impact on your partner (2, 3.4%)  Access to baby supplies (e.g. formula, diapers, wipes) (1, 1.7%)  Impact on your community (0, 0.0%)  Impact on close friends (0, 0.0%)  Access to food (0, 0.0%)  Access to mental health care (0, 0.0%)  Stress about other (open field) (0, 0.0%) |
| G1-01 | Impact on your child |
| G1-02 | Health concerns |
| G1-03 | Impact on your child |
| G1-04 | Financial concerns |
| G1-05 | Impact on family members (e.g. elderly parents) |
| G1-06 | General well-being due to social distancing and/or quarantine |
| G1-07 | Health concerns |
| G1-08 | Impact on your child |
| G1-09 | General well-being due to social distancing and/or quarantine |
| G1-10 | Health concerns |
| G1-11 | Health concerns |
| G1-12 | Impact on your partner |
| G1-13 | Impact on family members (e.g. elderly parents) |
| G1-14 | Impact on family members (e.g. elderly parents) |
| G1-15 | Impact on your child |
| G1-16 | Impact on your child |
| G1-17 | Impact on your partner |
| G1-18 | Impact on family members (e.g. elderly parents) |
| G1-19 | Impact on family members (e.g. elderly parents) |
| G1-20 | Health concerns |
| G1-21 | General well-being due to social distancing and/or quarantine |
| G1-22 | Impact on your child |
| G1-23 | Health concerns |
| G1-24 | Impact on your child |
| G1-25 | Access to baby supplies (e.g. formula, diapers, wipes) |
| G1-26 | Financial concerns |
| G1-27 | Impact on society |
| G1-28 | I am not stressed |
| G1-29 | Health concerns |
| G1-30 | Impact on your child |
| G2-01 | Health concerns |
| G2-02 | General well-being due to social distancing and/or quarantine |
| G2-03 | Impact on your child |
| G2-04 | Health concerns |
| G2-05 | General well-being due to social distancing and/or quarantine |
| G2-06 | Financial concerns |
| G2-07 | Impact on your child |
| G2-08 | Impact on your child |
| G2-09 | Impact on family members (e.g. elderly parents) |
| G2-10 | Impact on family members (e.g. elderly parents) |
| G2-11 | I am not stressed |
| G2-12 | Impact on society |
| G2-13 | Health concerns |
| G2-14 | Financial concerns |
| G2-15 | Health concerns |
| G2-16 | Impact on your child |
| G2-17 | Financial concerns |
| G2-18 | Impact on your child |
| G2-19 | Impact on society |
| G2-20 | Impact on family members (e.g. elderly parents) |
| G2-21 | Health concerns |
| G2-22 | Impact on family members (e.g. elderly parents) |
| G2-23 | Impact on your child |
| G2-24 | Impact on your child |
| G2-25 | I am not stressed |
| G2-26 | Impact on society |
| G2-28 | Health concerns |
| G2-29 | Impact on your child |
| G2-30 | Impact on your child |
